# The “Black-and-White Cookie” Sign – A Case Series of a Novel Ultrasonographic Sign in Gastric Outlet Obstruction

**DOI:** 10.5811/cpcem.2017.11.35890

**Published:** 2018-01-09

**Authors:** Allison Cohen, Mark Foster, Brendon Stankard, Maxine Owusu, Mathew Nelson

**Affiliations:** North Shore University Hospital, Department of Emergency Medicine, Manhasset, New York

## Abstract

Gastric outlet obstruction (GOO) is a rare condition occurring as a consequence of numerous processes that prevent gastric emptying. Presenting symptoms of GOO are non-specific and include nausea, vomiting, epigastric discomfort and decreased appetite. The diagnosis of GOO is often challenging. Emergency physicians must have a heightened awareness of GOO to ensure proper diagnosis and rapid treatment. Although the gold standard for diagnoses of GOO is endoscopy, many patients are identified by computerized tomography imaging. Point-of-care ultrasound (POCUS) is a rapid and non-invasive technique for evaluating patients in the emergency department. Previous literature has validated the use of ultrasound in diagnosing various intra-abdominal pathologies including bowel obstructions and appendicitis; however, there is limited research on evaluating gastric disease.^1^ We report three cases of GOO diagnosed with the “black-and-white cookie” sign on POCUS.

## INTRODUCTION

Gastric outlet obstruction (GOO) is a clinical entity occurring as a consequence of numerous processes that prevent gastric emptying. Previously it was hypothesized that GOO was most commonly caused by benign conditions like peptic ulcer disease; however, malignancy is now known to be responsible for most new cases.^2^ GOO is a known complication of metastatic disease, most often of the upper gastrointestinal tract. The most common causes are pancreatic and gastric malignancies. However, lymphomas, ampullary carcinomas, and biliary tract disease also play a significant role.^3^

Presenting symptoms of GOO are nausea and vomiting, but it can present with epigastric discomfort and decreased appetite. Endoscopy is the gold standard for diagnoses of GOO, but many patients are identified by computerized tomography(CT) imaging, which provides information about the underlying cause.^4^ Proposed treatments for obstruction include both surgical and nonsurgical options depending on the underlying cause and goals of care. Proposed treatment options include gastrojejunostomy with laparotomy or a laparoscopic approach and endoscopic stenting.^5^ We report three cases of malignant GOO diagnosed with point-of-care ultrasound (POCUS) in the emergency department (ED).

## CASE REPORT

### Case 1

**Image 1ab:**
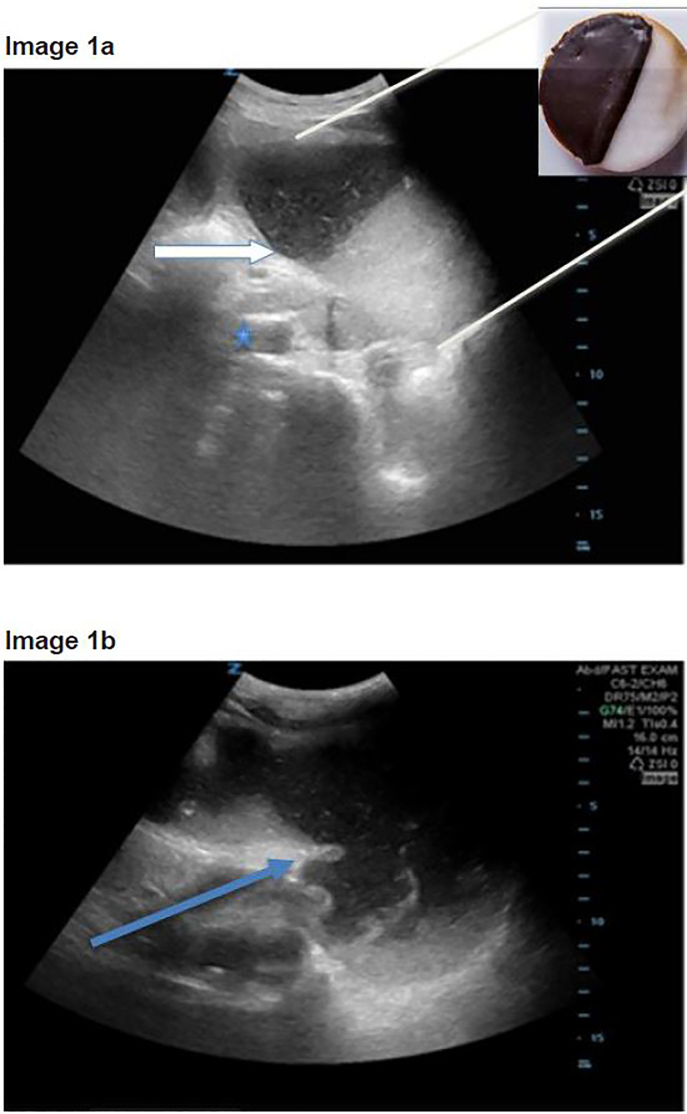
a) Sagittal orientation point-of-care ultrasound (POCUS) with “black-and-white cookie” sign seen by white arrow with division of stomach contents. Inferior vena cava (blue star) seen distal to the stomach; b) POCUS of dilated, fluid-filled stomach with thickened stomach wall (blue arrow).

A 72-year-old-female with a past medical history of hypertension, pulmonary embolism, and ovarian carcinoma presented to the ED with hypotension, vomiting and two syncopal episodes. Over the prior three weeks the patient reported diminished appetite and poor oral intake secondary to persistent nausea and vomiting. She denied any abdominal pain and reportedly was having normal bowel movements and flatus. Upon arrival to the ED, she had a blood pressure of 94/72mm Hg and a heart rate of 112 beats per minute. On physical exam, the patient was ill appearing and frail. Her abdomen was distended without peritoneal signs. In the ED, the patient remained hypotensive and therefore a POCUS was performed using the Rapid Ultrasound in Shock and Hypotension (RUSH) protocol.^6^ The ultrasound showed no evidence of cardiogenic shock, a flat inferior vena cava, and free intraperitoneal fluid. While attempting to visualize the splenorenal fossa, a distended stomach was visualized. (Image 1b)

The gastric contents layered in a meniscal fashion, appearing like a black-and-white cookie. (Image 1a) There was no evidence of dilated bowel or bidirectional peristalsis to suggest a bowel obstruction. With concern for a distended stomach and abdominal free fluid without further evidence of bowel obstruction, a CT was ordered to further evaluate for gastric obstruction. The CT showed a dilated, fluid-filled distal esophagus and stomach consistent with GOO. The obstruction was secondary to loculated ascites and peritoneal carcinomatosis from the patient’s metastatic ovarian cancer. A nasogastric tube was placed, draining more than two liters of stomach contents. The patient was admitted to the hospital for further evaluation. The gastroenterology, oncology and surgical services were consulted. After a family meeting the patient went home with hospice services.

CPC-EM Capsule

What we already know about this clinical entity?

Gastric outlet obstruction (GOO) is a rare clinical condition occurring as a consequence of numerous processes that prevent gastric emptying.

What makes this presentation of disease reportable?

In this case series, we describe how point-of-care ultrasound (POCUS) was used to demonstrate common findings in three separate patients presenting with GOO.

What is the major learning point?

In all three patients, the stomach had a very distinct appearance on POCUS resembling a black-and-white cookie. This appearance was caused by a division of solid particles and gastric secretions within the stomach cavity.

How might this improve emergency medicine practice?

With improved availability, POCUS is often being used as a key tool for evaluation of bowel pathology. Emergency providers need to have a heightened awareness of GOO to ensure proper diagnosis and treatment. Although computerized tomography remains the standard diagnostic modality, the use of POCUS could represent a promising imaging alternative.

### Case 2

**Image 2ab:**
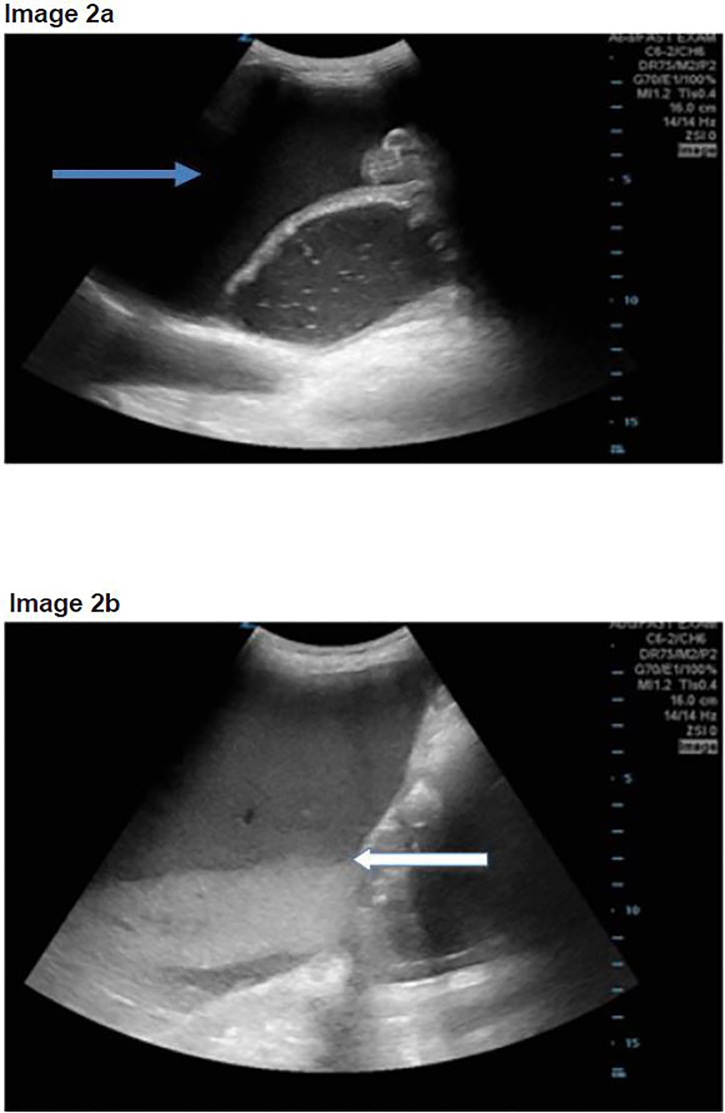
a) Point-of-care ultrasound (POCUS) with dilated stomach and surrounding intraperitoneal free fluid (blue arrow); b) POCUS with “black-and-white cookie” sign seen by white arrow with division of stomach contents.

A 71-year-old-male with a past medical history of coronary artery disease, hypertension, and newly diagnosed liver tumors presented to the ED with increased weakness, lower extremity edema, diminished appetite, nausea and vomiting. He denied any fever, abdominal pain or diarrhea. Upon arrival, the patient was found to be hypotensive with a blood pressure of 52/37mmHg, a heart rate of 80 beats per minute and oxygen saturating 100% on room air. On physical exam, the patient was ill appearing and with signs of acute distress and shock. The patient had dry mucous membranes, icteric sclera, clear lungs, lower extremity edema and a distended but soft abdomen. He was started on broad-spectrum antibiotics and given aggressive intravenous hydration. A POCUS demonstrated free intraperitoneal fluid (Image 2a), no evidence of interstitial pulmonary edema, and again a large, dilated stomach with a black-and-white cookie appearance (Image 2b), without evidence of small bowel obstruction. Although the patient denied any abdominal pain, the ultrasound results prompted the emergency physician (EP) to order a CT of the abdomen. The CT showed GOO with a dilated stomach with extrinsic compression of the duodenum due to mass effect from hepatomegaly and ascites. The patient was admitted to the medical intensive care unit (MICU) for blood pressure support, palliative care consult and further care. In the MICU a family meeting took place and the patient was made hospice care only. The patient expired four days later.

### Case 3

**Image 3:**
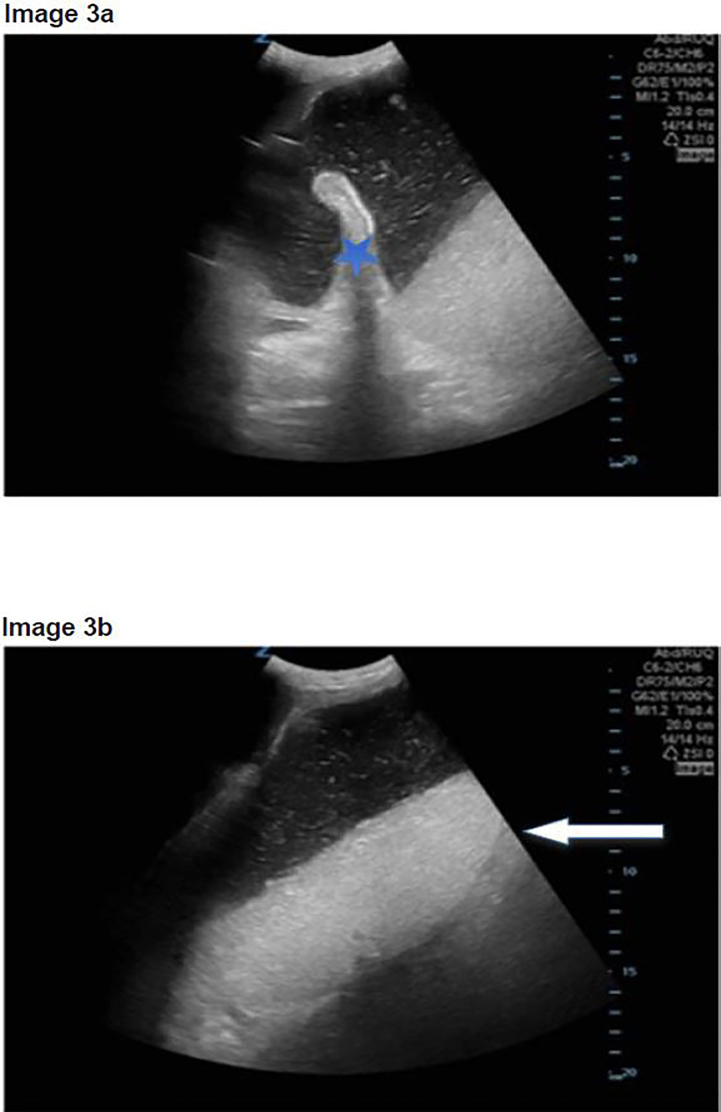
a) Point-of-care ultrasound (POCUS) with dilated stomach with visible stomach fold (blue star) and bidirectional flow of stomach contents; b) POCUS with “black-and-white cookie” sign seen by white arrow with division of stomach contents.

A 69-year-old-woman with a history of hypertension, breast cancer and cholangiocarcinoma with an indwelling percutaneous biliary drain presented to the ED after an episode of coffee ground emesis associated with acid reflux symptoms. The patient complained of constipation, decreased appetite and weight loss. She denied any hematochezia and reported a normal last bowel movement. In the ED, the patient was frail appearing with a blood pressure of 100/60 mm Hg and a heart rate of 105 beats per minutes. On physical examination, the patient was jaundiced with scleral icterus. The abdomen was not tender but distended and firm with hyperactive bowel sounds. A biliary drain was also present without surrounding erythema or visible signs of infection. A rectal exam was negative for blood or presence of melena. A POCUS was performed showing a dilated stomach (Image 3a) without further evidence of bowel obstruction. Again, the stomach had the black-and-white cookie appearance on POCUS (Image 3b).

A CT scan of the abdomen displayed a dilated stomach with abrupt caliber change secondary to a porta hepatis mass extending to the pancreas causing a GOO. A nasogastric tube was placed in the ED draining approximately 1,500ml of gastric contents. The patient was admitted to the surgical service for further management and gastroenterology consult. During her hospital stay the patient had a metal stent placed to treat the GOO. The patient improved and was discharged home with gastroenterology and oncology follow-up.

## DISCUSSION

POCUS is an invaluable tool to enhance decision-making in all patients and especially those that are critically ill. The incorporation of POCUS for patients with undifferentiated shock and hypotension is a well-validated tool to improve diagnostic accuracy and guide goal-directed treatments.^7^ Implementing protocols for goal-directed ultrasound with undifferentiated hypotension has been shown to increase physician accuracy and improve time to diagnosis.^8^ All three patients in this series received a RUSH protocol ultrasound.

While performing the Focused Assessment of Sonography in Trauma component of the exam and evaluating the splenorenal interface, a prominent, fluid-filled structure was visualized anterior to the spleen with bidirectional flow of internal contents, and was identified as the stomach. The ultrasound was performed while the patient was in the supine position, using a low frequency Zonare curvilinear probe. The transducer was placed on the patient’s left flank to obtain a coronal view of the splenorenal fossa. Once the left kidney was identified, the probe was fanned anteriorly to visualize a thin-walled, dilated, fluid-filled structure not containing plique circularis or haustra, which was identified as the stomach. As the gastric antrum is the most easily visualized portion of the stomach and is commonly found anterior to the left lobe of the liver and posterior to the pancreas, it can be viewed by placing the transducer in the epigastric region in a sagittal orientation.^9^

As a dilated stomach cavity is often present with both GOO and small bowel obstructions, it is critical that the remaining bowel be evaluated in order to differentiate the two entities. In all three patients, the stomach had a very distinct appearance on POCUS resembling a black-and-white cookie. This appearance was caused by a hyperechoic meniscal layer of gastric contents that contained an internal division of anechoic as well as hyperechoic areas. This division is secondary to solid particles and gastric secretions moving towards the more dependent portions of the stomach, while less dense materials and gas separate and move anteriorly.^10^ Sonographic findings that should prompt further evaluation for possible GOO include a dilated and enlarged gastric cavity in a patient without further evidence of a bowel obstruction.

Currently there is minimal literature on ED diagnoses of GOO using POCUS in adults. Stomach size is variable and dependent on many factors including timing of last meal. There is a paucity of research focusing on the use of POCUS in diagnosing and evaluating it for this pathology. A recent case report demonstrated the novel use of POCUS for identifying GOO; however, corroborating literature is limited.^11^ Anesthesia as well as gastroenterology studies have focused on the use of ultrasound for evaluating the stomach volume and wall thickness to evaluate for aspiration risk during intubation, gastric malignancies and gastric emptying disorders. Prior studies have also focused on the predictive value of gastric fluid volume for identifying various pathologies. One study hypothesized that patients with increased gastric fluid, with an area greater than 10cm^2^, had a greater likelihood of having either a gastric obstruction or duodenal ulcer.^12^ All three patients evaluated in this case series had distended-appearing, large-volume stomachs without any evidence of bowel obstruction on POCUS.

GOO can be a challenging diagnosis to make in the ED. Its presenting symptoms of nausea, vomiting and cachexia are nonspecific and mimic many other illnesses. It is expected that the incidence of GOO will increase, as malignancy is now the most common etiology of new diagnoses. EPs need to have a heightened awareness of GOO to ensure proper diagnosis and treatment. Although the CT still remains the standard diagnostic modality for GOO, the use of POCUS could represent a promising imaging alternative. EPs should have an elevated suspicion for GOO in patients with a history of nausea, vomiting, known malignancy with a distended stomach on POCUS without further evidence of bowel distention or obstruction. As the use of POCUS has continued to advance, its application to bowel pathology has become more recognized and validated. The use of POCUS for diagnosing GOO is a novel technique that requires further study, but it has the potential to allow for more rapid diagnosis and management of this cohort of patients.
